# Transdermal Fentanyl Patches Versus Patient-Controlled Intravenous Morphine Analgesia for Postoperative Pain Management

**DOI:** 10.5812/ircmj.11502

**Published:** 2014-05-05

**Authors:** Mohamad Hossein Ebrahimzadeh, Seyed Kamal Mousavi, Hami Ashraf, Rahil Abubakri, Ali Birjandinejad

**Affiliations:** 1 Department of Orthopedic Surgery, Orthopedic and Trauma Research Center, Mashhad University of Medical Sciences, Mashhad, IR Iran; 2 Orthopedic and trauma Research Center, Mashhad University of Medical Sciences, Mashhad, IR Iran

**Keywords:** Fentanyl, Morphine, Analgesia, Patients, Pain Management, Postoperative Period

## Abstract

**Background:**

Acute and severe pain is common in patients postoperatively and should be correctly managed. In the past years studies on preparing better postoperative pain control have resulted in development of postoperative pain management guidelines. Perhaps, one of the major improvements in managing postoperative pain is the development of the patient-controlled analgesia systems (PCA), especially through intra venous (IV), extradural and transdermal routes, which has resulted in marked improvements in acute postoperative pain management. Physicians administrate potent opioids for moderate to severe post-surgical pains. Morphine is the most commonly IV-PCA administrated analgesic. The fentanyl iontophoretic transdermal system (fentanyl ITS) is also another efficient option for pain management.

**Objectives:**

The aim of this study was to compare the analgesic effects of these two routine postoperative pain control systems.

**Patients and Methods:**

We enrolled 281 patients (224 males, 57 females) in this blind randomized controlled clinical trial, who had undergone an orthopedic surgery, with the mean age of 33.91 ± 14.45 years. Patients were randomly divided into two groups; in group A patients received IV-morphine PCA pump and in group B fentanyl transdermal patches were attached on patients’ arms. The severity of the pain was registered according to Visual Analogue Scale in specially designed forms by pain-trained nurses in two steps; first after the surgery and next before the beginning of analgesic effects. After 24 hours, the pain score was assessed again.

**Results:**

No significant difference was observed in mean pain intensity score at the first patient assessment. Mean pain intensity scores were also similar in both groups at the last measured time point (P > 0.05). Differential pain intensity scores, showing the impacts of analgesic system on the pain experience of the patients was also similar between fentanyl patches (6.48 ± 2.20) and morphine PCIA (6.40 ± 1.80). (P > 0.05) Mean patient satisfactory score (scale: 0–100) was also similar in both groups (P > 0.05). The percentage of patients, whose differential pain intensity scores at 24 hours reached our pain management goal was similar between fentanyl and morphine groups (P > 0.05). The percentage of patients with at least one adverse event was significantly higher in fentanyl group (P < 0.05). The most frequent adverse events were nausea, vomiting and itching. In none of the groups, no patient experienced serious adverse events related to the studied medications.

**Conclusions:**

Although both pain killing therapeutic regimens are safe and effective for postoperative pain management, regarding the easy usage of the patches, lower risk of abuse and cost-effectiveness in the Iranian market, it is recommended for use in Iranian hospitals and trauma centers and in countries with similar socioeconomic situations.

## 1. Background

Pain is a mysterious sense in human beings and one of the commonest reasons for visiting a doctor (1). It could completely disturb life quality of the patients. Therefore the pain should be controlled, especially in chronic or severe forms. Under-treatment or mistreatment of the pain is common and make patients visit different doctors seeking for relief (2, 3). Pain is responsible for billion hours work loss, dollars for medical costs and social and family problems (2, 4, 5). On the other hand, nowadays analgesic abuse is one of the medical and social challenges. Therefore, choosing and prescribing the best analgesic choice for every patient is important. The primary analgesic administration route for general use is oral, in most of the international pain guidelines (6), but it mainly depends on the patient situation, condition of analgesic abuse and severity of pain. For preparing better postoperative pain control, many studies conducted in the past years have resulted in development of post-op pain management guidelines (7-9).

Most of the analgesic regimens are intermittent, but pain is constant in post-surgical patients. Therefore it may lead to inadequate pain control (10, 11) or analgesic overdose. Perhaps, one of the major improvements in postoperative pain management is patient-controlled analgesia systems (PCA), (12) especially in IV, extradural and transdermal routes. This has resulted in marked improvements in acute postoperative pain management (13, 14). Physicians widely administrate potent opioids for moderate to severe post-surgical pain (15). Beside morphine, which is known as the most frequent IV administrated analgesic for PCA, other opioids including fentanyl, are also used (16). Fentanyl is a low molecular weight synthetic opioid, with high potency analgesic effect in intravenous route injection. According to IV doses, fentanyl is considered to have 50 to 100 times the potency of morphine (17). Due to the small molecule structure and high lipid solubility, fentanyl could be a good choice for transdermal use (18).

The fentanyl iontophoretic transdermal system (fentanyl ITS) is a self-programmed easy-applicable needle-free analgesic delivery way, designed for chronic and acute pain management, which could be helpful for postoperative adult patients. Although these patches provide the drug via the skin at a constant rate, the pharmacokinetic profile resembles IV fentanyl (18). Therefore it is reasonable to compare the analgesic efficacy and side effects of these two routine postoperative pain control systems.

## 2. Objectives

The aim of this study is to compare the analgesic effects of these two routine postoperative pain control systems.

## 3. Patients and Methods

We enrolled 281 patients (224 males, 57 females) in this blind randomized controlled clinical trial, with the mean age of 35.01 ± 14.49 years, after filling the inclusion criteria. They were fully informed and signed the ethical committee consent forms. Double blinding the study for the patients and all the staff involved was impossible, but our sampling, statistical analysis and medical history taking were performed by trained nurses who were completely blinded to therapeutic groups. All patients had undergone an orthopedic surgery in Shahid Kamyab Trauma Center and University Hospital, January 2011 to January 2012. Patients were randomly divided into two groups, for postoperative pain control.

**Figure 1.**
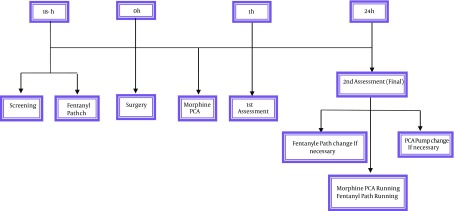
Study Protocol

In group A patients received IV-morphine PCA pump. Each pump were filled by 20 mg morphine, diluted in 100 mL normal saline and set on 1 mg/h infusion rate. One bolus dose could be added by the patient if he/she felt undesirable pain. In group B fentanyl transdermal patches with total dose of 25 μg were used. Regarding the delayed action of the patches, they were attached upon the patients arm 18 h before the operation (Figure 1).

The severity of the pain was registered, according to Visual Analogue Scale in especially designed forms, by pain-trained nurses in two steps; first after the surgery and then before the beginning of analgesic effects. No other analgesic drug was used during this time. Therefore, 24 hours after the surgery pain was assessed again. According to the surgery type, patients were enrolled in three categories: small surgeries on the fingers, and skin grafts were considered as the minor surgeries, moderate surgeries involved fractures of the forearm and foreleg and injury debridement and major surgeries involved pelvic surgeries (DHS, interlock), surgeries on femur, cervical and lumbar vertebrae, shoulder, amputations and prosthesis. The statistical analysis was done with SPSS software version 16.0, t-student and k-square test. Our profession staffs in vital statistics analysis were completely blinded to the therapeutic groups.

## 4. Results

A total of 281 patients were enrolled into the study (fentanyl ITS group, n = 138, morphine PCIA group, n = 143). The mean age of the patients was 35.93 ± 15.73 years in fentanyl group and 34.13 ± 13.18 years in morphine PCIA patients (P = 0.296). No significant difference was observed between the two groups regarding patient characteristics (Table 1). The distribution of types of the surgery was similar between groups (five patients in minor, 77 in moderate and 61 in major surgeries in morphine PCA group vs. eight in minor, 61 in moderate, and 69 in major surgeries in fentanyl TCI, P = 0.229) (Table 1). No patient, neither in the fentanyl ITS group, nor in the morphine PCIA group received a spinal anesthetic or any extra analgesic with his/her defined protocol.

**Table 1.** Patients Characteristics ^a^

	Morphine	Fentanyl	Total	P value
**Age, y**	34.13 ± 13.18	35.93 ± 15.73	35.01 ± 14.49	0.296
**Median**	30	32	32.00	
**Range **	16-69	14-80	14-80	
**Other internal diseases**	29 (20.3)	40 (29.0)	69 (24.6)	0.090
**Multiple trauma**	34 (23.8)	27 (19.6)	61 (21.7)	0.392
**Surgery type**				0.229
Minor	5 (3.5)	8 (5.8)	13 (4.6)	
Moderate	77 (53.8)	61 (44.2)	138 (49.1)	
Major	61 (42.7)	69 (50.0)	117	
**Addiction**				0.102
Addicted	42 (29.4)	54 (39.1)	96 (34.2)	
Non addicted	101 (70.6)	84 (60.9)	185 (34.2)	
**Cigarette smoking**				0.496
Smoker	74 (51.7)	77 (55.8)	151 (53.7)	
Non smoker	69 (48.3)	61 (44.2)	130 (46.3)	
**Analgesic use**	37 (25.9)	43 (31.2)	80 (28.5)	0.326

^a^ Data are presented in No. (%).

Mean ± SD of patient satisfactory score (scale: 0–100), at the last assessment after the first 24 hours were similar in both groups (76.95 ± 1.78 for fentanyl ITS and 73.57 ± 18.49 for morphine PCIA, P = 0.147) (Table 2).

**Table 2.** Patient Satisfactory and Pain Scores Between Therapeutic Groups^a, b^

	Morphine PCIA	Fentanyl ITS	P value
**Patient satisfactory score**	73.57 ± 18.48	76.96 ± 20.61	0.147
**1^st^** ** pain intensity score**	8.56 ± 1.43	8.36 ± 1.50	0.252
**2^nd^** ** pain intensity score**	2.15 ± 1.55	1.87 ± 1.69	0.153
**Differential pain intensity scores**	6.40 ± 1.80	6.48 ± 2.20	0.717

^a^ Data are presented as Mean ± SD.

^b^ Abbreviations: ITS, iontophoretic transdermal; PCIA, system patient-controlled analgesia systems.

The percentages of patients who reported satisfactory ratings of good or excellent, after the first 24 hours were 36.2% and 45.7%, respectively for the fentanyl ITS group vs. 42% and 42% for the morphine PCIA group. The difference was not significant (P = 0.746). There was no significant difference in mean pain intensity score, observed at the first patient assessment (8.56 ± 1.43 in morphine vs. 8.36 ± 1.50, P = 0.252). Mean pain intensity scores were also similar in both groups at the last measured time point (2.15 ± 1.55 in morphine vs. 1.87 ± 1.69 in fentanyl, P = 0.153). Differential pain intensity scores which show the impact of analgesic system on the pain experience of the patients, was also similar between patients with fentanyl patches (6.48 ± 2.20) and morphine PCIA (6.40 ± 1.80) patients (P= 0.717).

The percentage of patients whose differential pain intensity score reached our pain management goal, in 24 hours, was similar between fentanyl and morphine groups (71.7 vs. 74.8%; P = 0.559). The incidence of common adverse events was comparable between the two groups. The percentage of patients having at least one adverse event was significantly higher in fentanyl group (2.9 vs. 18.1%, respectively; P = 0.00). The most frequent adverse events were nausea and vomiting (0.152% of fentanyl ITS patients and 0.007% of morphine PCIA patients) and itching (0.028% of fentanyl ITS patients and 0.021% of morphine PCIA patients). No patient experienced serious adverse events (SAEs), related to the studied medications like ileus, somnolence, hernia or hypoventilation. Respiratory function was the primary safety measurement. No patient, neither in the fentanyl ITS group, nor in the morphine PCIA group experienced clinically relevant respiratory depression (bradypnea < 8 bpm and excessive sedation) (Table 3).

**Table 3.** Patient Complications Between Therapeutic Groups

	No	Nausea & Vomiting	Sedation	Urinary Retention	Constipation	Itching
**Morphine**	134	1	0	0	0	3
**Fentanyle**	113	21	4	4	0	4
**Total**	247	22	4	4	0	7

## 5. Discussion

As judged by patient satisfactory scores, there was no statistically significant difference between two therapeutic regimens. The other efficacy variable, pain intensity score, confirmed the primary satisfactory data. In recent two decades, there have been many reports about safety and efficacy of PCA in post-surgery setting with potent opioids, especially with morphine or fentanyl (19-23).

Our study supports the results of previous trials that demonstrated fentanyl ITS to be effective on postoperative pain (12, 24, 25), at least as much as standard morphine PCIA regimen in patients who are not drug-dependent (26-28). Both approaches seem to be safe and highly effective. Although, different withdrawal rates were assessed for inadequate analgesia in Viscusi et al. study, as more patients in the fentanyl ITS group compared with the morphine IV PCA group (29), we had no patient who discontinue the study because of uncontrolled pain. Although there were some patients exiting the study due to the staff technical faults.

The inherent safety of PCIA is that the dosing frequency is controlled by the patient as needed for pain relief, reducing the possibility of overdose, as pain requirements are met. A meta-analysis of 15 randomized controlled studies showed that patients using PCA postoperatively, obtained significantly better pain relief than those using intramuscular analgesia, without increasing the adverse effects (30). This safety is maybe more prominent in fentanyl ITS. Panchal et al. believed that systemic related events, especially those related to needle injection like infiltration at the catheter site, pain at injection site and incorrect dosing or programming, occur more frequently in the morphine group. Also, less analgesic gap and interruption are reported in management of postoperative pain with fentanyl ITS vs. morphine pumps (31). Moreover, morphine and its metabolites can accumulate in patients with renal failure and be potentially toxic. Morphine also stimulates histamine release, increasing the risk of hypotension (32)., but there was not any evidence on histamine release in as a result of fentanyl use (33, 34), which makes it a proper option for pain management in patients with renal failure (35).

On the other hand, the most prevalent complications like itching, erythema, edema and discoloration, reported in literature about the fentanyl ITS group are related to the method of pain control (25). Panchal et al. in their study reported more device failures with fentanyl ITS (31). Another important point about analgesic prescription for postoperative pain management is patient and personnel cooperation and easy care factors. In Grond et al. (25) study was reported a significantly (P = 0.001) more favorable ease of care score, rated by the patients. Nurses and physiotherapists also reported care giving, significantly easier, less bothersome and more time-consuming in fentanyl ITS, compared to morphine PCIA (25) and although, the level of satisfaction between groups was the same (P = 0.804), nurses and physiotherapists reported higher (more favorable) mean (SEM) satisfaction ratings in the fentanyl ITS group.

To sum up, although both anti-pain therapeutic regimens are safe and effective for postoperative pain management, regarding the easy usage of the fentanyl patches, lower risk of abuse and cost-effectiveness in the Iranian market, its use is recommended in Iranian hospitals and trauma centers and countries with similar socioeconomic situations.
